# Sulfated archaeol glycolipids: Comparison with other immunological adjuvants in mice

**DOI:** 10.1371/journal.pone.0208067

**Published:** 2018-12-04

**Authors:** Bassel Akache, Felicity C. Stark, Yimei Jia, Lise Deschatelets, Renu Dudani, Blair A. Harrison, Gerard Agbayani, Dean Williams, Mohammad P. Jamshidi, Lakshmi Krishnan, Michael J. McCluskie

**Affiliations:** 1 National Research Council Canada, Ottawa, Ontario, Canada; 2 Department of Biochemistry, Microbiology and Immunology, University of Ottawa, Ottawa, Ontario, Canada; Midwestern University, UNITED STATES

## Abstract

Archaeosomes are liposomes traditionally comprised of total polar lipids (TPL) or semi-synthetic glycerolipids of ether-linked isoprenoid phytanyl cores with varied glyco- and amino-head groups. As adjuvants, they induce robust, long-lasting humoral and cell-mediated immune responses and enhance protection in murine models of infectious disease and cancer. Traditional total polar lipid (TPL) archaeosome formulations are relatively complex and first generation semi-synthetic archaeosomes involve many synthetic steps to arrive at the final desired glycolipid composition. We have developed a novel archaeosome formulation comprising a sulfated disaccharide group covalently linked to the free sn-1 hydroxyl backbone of an archaeal core lipid (sulfated S-lactosylarchaeol, SLA) that can be more readily synthesized yet retains strong immunostimulatory activity for induction of cell-mediated immunity following systemic immunization. Herein, we have evaluated the immunostimulatory effects of SLA archaeosomes when used as adjuvant with ovalbumin (OVA) and hepatitis B surface antigen (HBsAg) and compared this to various other adjuvants including TLR3/4/9 agonists, oil-in-water and water-in-oil emulsions and aluminum hydroxide. Overall, we found that semi-synthetic sulfated glycolipid archaeosomes induce strong Ag-specific IgG titers and CD8 T cells to both antigens. In addition, they induce the expression of a number of cytokines/chemokines including IL-6, G-CSF, KC & MIP-2. SLA archaeosome formulations demonstrated strong adjuvant activity, superior to many of the other tested adjuvants.

## Introduction

Adjuvants are critical components of modern day vaccines, enabling protection against multiple pathogens through their ability to enhance immune responses to the inherently weak disease-associated antigens found in subunit vaccines that lack the immunostimulatory molecules present in live or attenuated vaccines. Development of a clinically successful vaccine relies on the selection of an adjuvant with i) the appropriate degree of immune response magnification; ii) the required Th1 vs. Th2-based immune bias or induction of CD8 T cells; iii) relatively simple formulation and administration process; as well as iv) good general safety including minimal local reactogenicity.

Aluminum salts (alum) are the original adjuvant, first administered to human patients in the early 1930s as a component of toxoid vaccine formulations against *Clostridium tetani* and *Clostridium diphtheria* infections. The inclusion of alum was based on the observation that it could induce higher antibody responses to vaccine antigens *in vivo* [1]. Due to their clinical efficacy, acceptable safety profile and wide-spread use, alum salts were the only adjuvant approved for human vaccines for 70 years thereafter. As such, all newly developed adjuvanted vaccines up till the 1990s included aluminum salts (e.g., vaccines against hepatitis A & B viruses, human papilloma virus, pneumococcus and meningococcus) [2]. This was despite the lack of an in-depth understanding of its mechanism of action at the time, which is now widely thought to be due a combination of an antigen depot effect and immunomodulatory activity through the NLRP3 inflammasome [3].

Although used in human vaccines for over 70 years, alum was insufficiently immunogenic to induce protection in all patient populations and to more challenging diseases. For example, while the alum-adjuvanted Hepatitis B surface antigen (HBsAg) vaccine EngerixB has a high responder rate in the general patient population, certain subpopulations such as the elderly and diabetics have much lower response rates and can remain susceptible to infection despite vaccination [4;5]. While alum can enhance antibody responses to a vaccine antigen, it has been shown to be a weak inducer of cellular immune responses thought to be necessary to better fight certain intracellular pathogens (viral & bacterial) and cancer-based diseases [1]. In addition, alum has been associated with the formation of granulomas, which may lead to persistent itching subcutaneous nodules and allergy to aluminium [6]. The need for more effective adjuvants coupled with major advancements in our understanding of the immune system, led to the development and inclusion of novel adjuvants belonging to various molecule classes with differing mechanisms of action in FDA and/or EU-approved vaccines. For example, the squalene-based oil-in-water emulsions AS03 and MF59 have been approved in the influenza vaccines Pandemrix and Fluad, respectively [2;7]. TLR agonists such as Monophosphoryl lipid A (MPL) and CpG are key components of the more potent second-generation Hepatitis B vaccines Fendrix and Heplisav-B, respectively [8]. These vaccines are more suitable for specific patient populations such as diabetics that generally respond poorly to the aluminum adjuvanted vaccine. The newly approved Shingles vaccine, Shingrix, is adjuvanted with AS01, an adjuvant system that combines two immunostimulatory molecules, MPL & the saponin QS-21, with liposomes, and has been shown to generate stronger and longer-lasting responses increasing its efficiency in the elderly population [9].

Archaeosomes are a novel class of potential vaccine adjuvants shown to induce strong cellular and humoral immune responses to antigens *in vivo*. Archaeosomes consist of liposomes formed with lipids containing the characteristic ether linkages of archaea between the carbon chains and the glycerol backbone. Vaccination with antigen encapsulated within archaeosomes formed with the total polar lipids (TPL) from various archaea species (e.g. *Methanobrevibacter smithii*) has been shown to enhance the immunogenicity of a broad range of antigens. In preclinical studies, they were able to mediate protection from pathogens such as *Listeria monocytogenes* as well as solid and metastatic tumors [10;11]. More recently, semi-synthetic glycerolipids such as sulfated S-lactosylarchaeol (SLA) either alone or mixed with uncharged glycolipid (lactosylarchaeol, LA) have been used to form archaeosomes capable of enhancing antigen immunogenicity [12]. These semi-synthetic formulations offer many advantages such as homogeneity/purity, consistency of manufacturing and/or ease of synthesis when compared to previous TPL or other complex semi-synthetic archaeosome formulations. In addition, they have been shown to be well-tolerated with a favorable safety profile when administered intramuscularly *in vivo*, while stimulating strong cytokine secretion, immune cell recruitment and antigen uptake at the vaccination site [13]. In preliminary pre-clinical studies, certain TPL archaeosome-adjuvanted vaccines were shown to be superior to those adjuvanted with conventional liposomes or alum when administered intraperitoneally or subcutaneously [14]. However, the adjuvanticity of the novel semi-synthetic archaeosomes has not yet been compared to other adjuvant types. Herein, we have evaluated the immunostimulatory effects of SLA archaeosomes when used as adjuvant with ovalbumin (OVA) and hepatitis B surface antigen (HBsAg) and compared this to commercially available adjuvants including TLR3/4/9 agonists, oil-in-water and water-in-oil emulsions and aluminum hydroxide.

## Materials & methods

### Ethics statement

Mice were maintained at the small animal facility of the National Research Council Canada in accordance with the guidelines of the Canadian Council on Animal Care. All procedures performed on animals in this study including anesthesia by isoflurane and euthanasia by cervical dislocation were in accordance with regulations and guidelines reviewed and approved in animal use protocol 2016.08 by the National Research Council Human Health Therapeutics Ottawa Animal Care Committee.

### Antigens

Endograde endotoxin-free ovalbumin (OVA; Hyglos, Bernried am Starnberger See, Germany) and recombinant Hepatitis B surface antigen (HBsAg; Subtype adw; Fitzgerald Industries International, Acton, MA, USA) from yeast were used.

### Adjuvants

*Halobacterium salinarum* (ATCC 33170) was grown, total polar lipids extracted from the resulting biomass were hydrolyzed, and archaeol core purified as previously described [15]. Structural identity and purity of archaeol was confirmed by thin layer chromatography, NMR spectroscopy and negative-ion fast atom bombardment mass spectrometry to be >90%. Thereafter, lactosylarchaeol (LA; *β*-d-Gal*p*-(1,4)-*β*-d-Glc*p*-(1,1)-archaeol) and sulfated lactosylarchaeol (SLA; 6'-sulfate-*β*-d-Gal*p*-(1,4)-*β*-d-Glc*p*-(1,1)-archaeol) were synthesized as reported previously [16;17] and tested with Endosafe cartridge-based Limulus amebocyte lysate test (Charles River Laboratories, Charleston, SC) to confirm lack of endotoxin contamination. Archaeosomes were formed by hydrating 20–30 mg dried lipid (SLA alone or 1:1 molar ratio of SLA/LA) at 40°C in 10 mg/mL OVA or ~3 mg/mL HBsAg (in water). Vesicle size was reduced to about 50–150 nm diameter by brief sonication or high pressure homogenization and the dry weight determined on an aliquot. Non-entrapped antigen was removed by centrifugation (200,000 x g max for 120 minutes) from 7 ml of water followed by 2 washes in water. Vesicle pellets were re-suspended, passed through a Millipore filter and formulated in phosphate-buffered saline (PBS; Millipore Sigma, Oakville, ON, Canada). Quantification of antigen loading was done through densitometry on the protein band following SDS polyacrylamide gel electrophoresis as previously described [18]. Loading of synthetic archaeosomes with antigens was also determined using SDS Lowry with standard curves prepared for the respective antigen (for OVA-based formulations only). Loading was based on μg protein/mg dry weight of lipid, and ranged from 4–9 and 10–116 μg /mg for HBsAg and OVA formulations, respectively (Table 1). Average diameters based on Intensity and Zeta potentials were measured using a Malvern Nano Zetasizer with a He/Ne laser (Spectra Research Corp., ON, Canada). The average diameters and zeta potentials ranged from 92–266 nm and -38 to -82 mV, respectively. Batches 1 and 2 were used for the vaccine and local inflammation studies, respectively (see below). All batches fall within acceptable parameters previously shown to enhance antigen immunogenicity.

**Table 1:** Particle size, Zeta potential and antigen:Lipid ratios of archaeosome formulations.

Archaeosome Formulation	Batch	Average diameter (nm)	Zeta potential (mV)	Antigen: Lipid ratio (μg/mg)
SLA-OVA	1	231	-50	77.2
	2	93	-67	15.6
SLA/LA-OVA	1	266	-38	116
	2	111	-72	10.1
SLA-HBsAg	1	173	-74	8.3
	2	103	-69	4.1
SLA/LA-HBsAg	1	153	-76	9.3
	2	94	-67	6.1

The immunostimulatory effects of archaeosomes composed of SLA or SLA/LA were compared against a panel of commercially available adjuvants at the indicated dose levels: i) Poly(I:C) (TLR3 agonist—Polyinosine-polycytidylic acid (HMW) VacciGrade, 40 μg, Invivogen, San Diego, CA, USA); ii) MPLA (TLR4 agonist—monophosphoryl Lipid A from *S*. *minnesota* R595 VacciGrade, 10 μg, Invivogen); iii) CpG (TLR9 agonist—ODN 1826 VacciGrade, Class B, murine, 10 μg, Invivogen); iv) Alum (Alhydrogel “85”, aluminum hydroxide, 40 μg Al^3+^, Brenntag Biosector, Frederikssund, Denmark); v) Addavax (1:1 v/v mixture squalene-oil-in-water emulsion, Invivogen); and vi) Montanide 720 (3:7 v/v water-in-oil emulsion, vegetable-grade oleic acid, Seppic, Paris, France). If necessary, formulations were diluted to the proper concentration with 0.9% saline solution (Invivogen) and stored at 4°C until administration to mice. Adjuvant dose levels were as recommended by the manufacturer and based on data from previous studies. All adjuvants were formulated as per their respective manufacturer’s instructions.

### Animals

6–8 week old female C57BL/6 and BALB/c mice were obtained from Charles River Laboratories (Saint-Constant, Canada) and used for OVA and HBsAg immunization, respectively. Animals were monitored for adverse clinical signs (such as piloerection, dehydration, hunched posture, labored breathing and reduced mobility) immediately following vaccination and routinely throughout the course of the study.

### Immunization of mice

Mice (n = 10/group) were immunized by IM injection (50 μL) into both the left and right tibialis anterior (T.A.) muscles (OVA immunizations) or the left T.A. muscle only (HBsAg immunizations) on Days 0 and 21 with a total dose of 20 μg OVA or 2 μg HBsAg alone or formulated with the various adjuvants as described above. Mouse strains, antigen and adjuvant doses for each antigen were determined to be optimal based on previous studies conducted in our laboratories or based on manufacturer’s recommendation.

Animals were bled on Days 20 & 28 and recovered serum was used for quantification of antigen specific IgG antibody levels. On Day 27, Carboxyfluorescein succinimidyl ester (CFSE)-stained target cells (as described below) diluted in Hank’s balanced salt solution (HBSS; GE Life Sciences, Chicago, IL, USA) to a final volume of 200 μL were injected into the retro-orbital plexus to assess antigen-specific *in vivo* cytolytic killing. Spleens were collected on Day 28 for elucidation of cellular immune responses by IFN-γ ELISpot or *in vivo* cytolytic activity assay.

### Anti-OVA/HBsAg Ab ELISA

The levels of anti-OVA or anti-HBsAg Abs in mouse serum were quantified by ELISA using 96–well high-binding ELISA plates (Thermo Fisher Scientific, Waltham, MA, USA) coated overnight at room temperature (RT) with 100 μL of 10 μg/mL OVA (Sigma-Aldrich, St. Louis, MO, USA) in PBS or 1 μg/mL HBsAg (Fitzgerald Industries International) in 16 mM sodium carbonate/ 34mM sodium bicarbonate buffer, pH 9.6 (Sigma-Aldrich). Plates were washed five times with PBS/0.05% Tween20 (PBS-T; Sigma-Aldrich), and then blocked for 1 hour at 37°C with 200 μL 10% fetal bovine serum (Thermo Fisher Scientific) in PBS or carbonate/bicarbonate buffer for OVA and HBsAg, respectively. After the plates were washed five times with PBS-T, 3.162-fold serially diluted samples in PBS-T with 10% fetal bovine serum were added in 100 μL volumes and incubated for 1 hour at 37°C. After five washes with PBS/0.05% Tween 20 (Sigma-Aldrich), 100 μL of goat anti-mouse IgG, IgG1, IgG2a or IgG2c-HRP (1:4000, Southern Biotech, Birmingham, AL USA) was added for 1 hour at 37°C. After five washes with PBS-T, 100 μL/well of the substrate o-phenylenediamine dihydrochloride (OPD, Sigma-Aldrich) diluted in 0.05 M citrate buffer (pH 5.0) was added. Plates were developed for 30 minutes at RT in the dark. The reaction was stopped with 50 μL/well of 4N H_2_SO_4_. Bound IgG Abs were detected spectrophotometrically at 450 nm. Titers for IgG in serum were defined as the dilution that resulted in an absorbance value (OD 450) of 0.2 and were calculated using XLfit software (ID Business Solutions, Guildford, UK).

### ELISpot

The levels of OVA or HBsAg specific T cells were quantified by ELISpot using a mouse IFN-γ kit (Mabtech Inc., Cincinnati, OH, USA). Splenocytes were isolated in RPMI media (Thermo Fisher Scientific) containing 10% FBS (Thermo Fisher Scientific), 1% penicillin/streptomycin (Thermo Fisher Scientific), 1% glutamine (Thermo Fisher Scientific) and 55 μM 2-Mercaptoethanol (Thermo Fisher Scientific). For OVA, 3x10^5^ cells were stimulated per well with peptides (OVA_257-264_: SIINFEKL or OVA_323-339_: ISQAVHAAHAEINEAGR (JPT Peptide Technologies GmbH, Berlin, Germany) or whole protein (Sigma-Aldrich) at a concentration of 2 and 10 μg/mL, respectively. For HBsAg, 4x10^5^ cells were stimulated per well with peptide (HBsAg_28-39_: IPQSLDSWWTSL, JPT Peptide Technologies GmbH) or whole protein (Fitzgerald Industries International) at a concentration of 2 and 5 μg/mL, respectively. These peptides correspond to well-recognized T cell epitopes in mice [19–21]. Final volume per well was 0.2 mL. Cells were also incubated without any stimulants to measure background responses. Plates were incubated for ~20 hours at 37°C with 5% CO_2_, at which point the plates were washed and developed according to the manufacturer’s instructions. AEC substrate (Becton Dickenson, Franklin Lakes, NJ, USA) was used to visualize the spots. Spots were counted using an automated ELISpot plate reader (BIO-SYS GmbH, Karben, Germany). Animals with high background responses (200 SFC/10^6^ splenocytes with media alone) were excluded from analysis.

### Assessment of *in vivo* cytolytic activity

*In vivo* cytolytic activity in mice was enumerated as described previously [22]. Briefly, vaccinated recipient mice were injected with an equal mixture of target cells (CFSE high: incubated with 50 μM CFSE) and non-target cells (CFSE low: incubated with 50 nM CFSE). Prior to labeling with CFSE (Thermo Fisher Scientific), target cells were pulsed with 10 μg/mL CD8+ T cell specific peptides (SIINFEKL for OVA experiments and IPQSLDSWWTSL for HBsAg experiments, JPT Peptide Technologies GmbH) and non-target cells were uncoated. The percentage of *in vivo* killing was determined approximately 20 h later on Day 28. Spleens were collected and the number of CFSE high and CFSE low cells quantified by flow cytometry on a BD LSRFortessa flow cytometer (Becton Dickenson). The survival proportions of target cell/non-target cell proportions as compared to naïve mice were then calculated to determine the cytolytic activity.

### Cytokine/chemokine levels in muscle

OVA and HBsAg vaccine formulations containing similar quantities of antigen and/or adjuvant as above were injected in a volume of 50 μL i.m. in the left T.A. of C57BL/6 and BALB/c mice, respectively (n = 3). Control mice were injected with 50 μL of PBS. Mice were euthanized 6 hours following injection to enable collection of the injected T.A. muscle which was frozen on dry ice. 500 μL of tissue protein extraction buffer (T-PER; Thermo Fisher Scientific) containing protease inhibitors (Complete Protease Inhibitor Cocktail; Roche Diagnostics, Basel, Switzerland) was added to the muscle tissues prior to homogenization using a Precellys lysing kit (Bertin Technologies, Versailles, France). Protein lysates were collected following centrifugation at 15,000 rpm for 10 min at 4°C. The levels of 17 different cytokines/chemokines in the lysates were determined by Luminex assay using a custom MILLIPLEX MAP Magnetic Bead Panel (Millipore, Billerica, MA, USA) according to manufacturer’s instructions. The cytokines/chemokines/growth factors analyzed were IFN-γ, IL-1β, IL-2, IL-4, IL-5, IL-6, IL-10, IL-17, TNF-α, G-CSF, GM-CSF, IP-10, KC, MCP-1, MIP-1α, MIP-1β and MIP-2, and were selected based on their roles in immune responses and previous studies conducted with these adjuvants. The concentration of these analytes was calculated using the MILLIPLEX Analyst software (Millipore). The protein concentration in the lysates was determined using the Pierce BCA protein assay kit (Thermo Fisher Scientific) and used to normalize the cytokine/chemokine levels per sample as pg/mg.

### Statistical analysis

Data were analyzed using GraphPad Prism (GraphPad Software, San Diego, CA). Statistical significance of the difference between groups was calculated by one-way analysis of variance (ANOVA) followed by post-hoc analysis using either Dunnett's (comparison with control unadjuvanted group) or Tukey's (comparison between all groups) multiple comparison tests. Antibody titers and cytokine levels were log transformed prior to statistical analysis. For all analyses, differences were considered to be not significant with p > 0.05. Correlation between data sets was also determined using GraphPad Prism by calculating the Pearson correlation coefficient. Raw data used to generate the various figures and tables below are included in S1 Dataset.

## Results

### Humoral response to OVA vaccine formulations in mice

**Fig 1:**
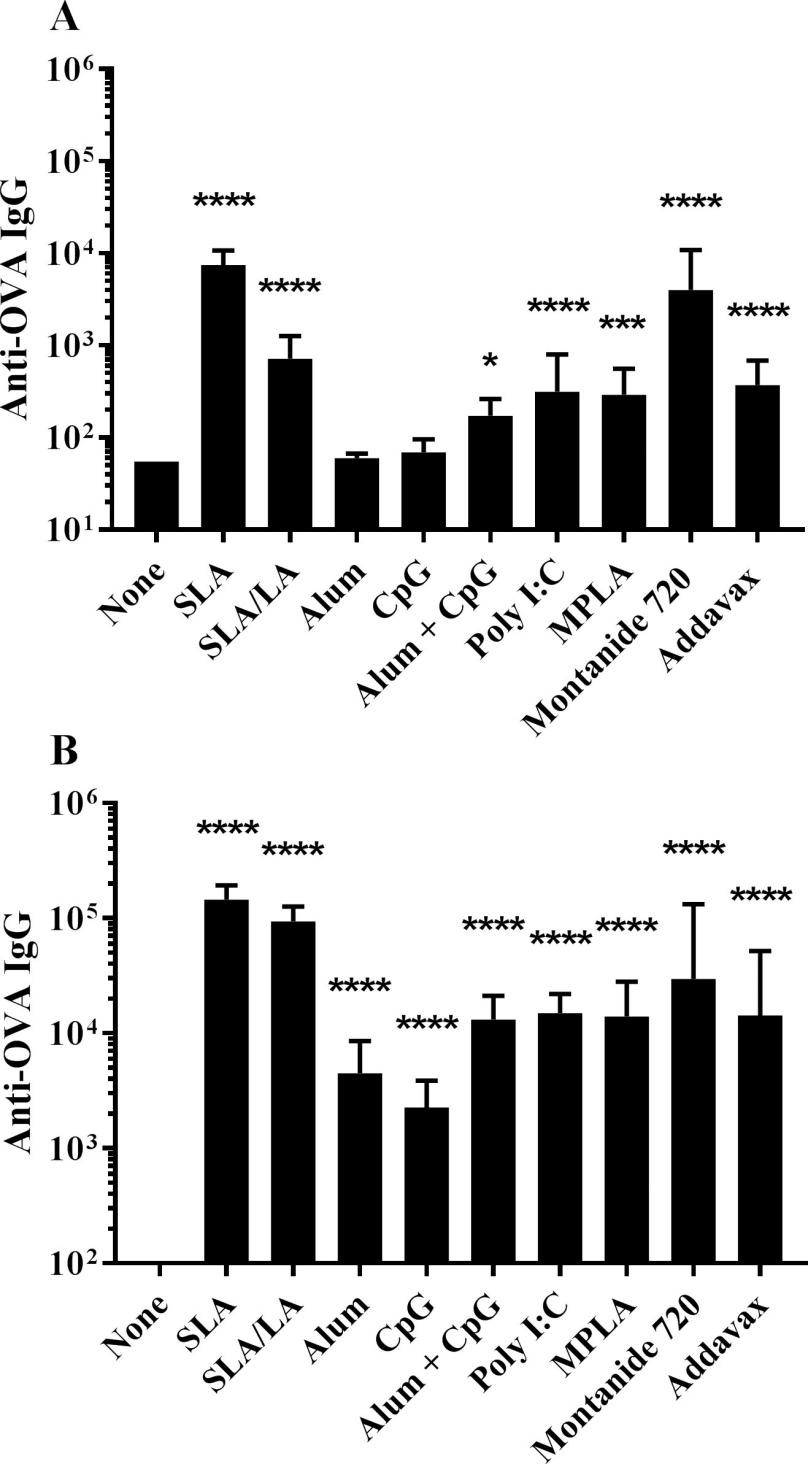
Anti-OVA IgG titers in immunized mice. C57BL/6 mice (n = 10/group) were immunized i.m. with ovalbumin (20 μg) with or without adjuvant on days 0 and 21. Animals were bled on Day 20 (Panel A) and 28 (Panel B) with serum analyzed for anti-OVA IgG Abs by ELISA. Grouped data is presented as geometric mean titer (GMT) + 95% Confidence Interval (CI). *: p<0.05, ***: p<0.001 & ****: p<0.0001 for various groups receiving adjuvanted formulations when compared to unadjuvanted control group by one-way ANOVA followed by Dunnett's multiple comparisons test.

C57BL/6 mice were immunized on Days 0 and 21 with OVA alone or in combination with different adjuvants including two separate archaeosome based formulations (SLA and SLA/LA), aluminum hydroxide (alum), the TLR agonists CpG (TLR9), Poly I:C (TLR3) and MPLA (TLR4), the water-in-oil emulsion Montanide 720 and the squalene-based oil-in-water emulsion Addavax (an MF59 mimetic). A combination of alum/ CpG as adjuvant was also tested. Anti-OVA IgG titres were assessed on Day 20 (20 days post single vaccine dose) and on Day 28 (7 days post second vaccine dose). Following a single administration, OVA in combination with all of the tested adjuvants, except for alum and CpG alone, induced anti-OVA IgG titers greater than those observed with OVA alone (Fig 1A). At day 20, highest anti-OVA IgG titers were induced with OVA plus SLA or Montanide 720 (p<0.0001 vs. unadjuvanted group) with geometric mean titers (GMT) (upper & lower 95% confidence interval (CI)) of 7498 (5238 & 10,734) and 3990 (1467 & 10,855), respectively. In addition, the titers obtained with OVA+ SLA or Montanide 720 were significantly higher than those generated with all tested adjuvants including SLA/LA (p<0.0001). Alum or CpG alone as adjuvant did not induce significant antigen-specific IgG titers following a single immunization, with only 3/10 mice in each group showing detectable levels of anti-OVA IgG.

As expected, higher levels of anti-OVA IgG were measured in serum following a second vaccination (Fig 1B). The greatest fold-increase was seen with SLA/LA+OVA, where GMT increased ~130-fold from Day 20 to 28. In contrast, mice immunized with Montanide 720+OVA only showed a ~3.6-fold increase in titers after boost. When combined with OVA, all adjuvant formulations induced Ag-specific Ab titers greater than those observed with OVA alone at Day 28 (p<0.0001). The relative ranking of the various adjuvanted vaccine formulations to induce anti-OVA IgG was similar to that observed pre-boost, with SLA and Montanide 720-adjuvanted OVA formulations generating GMT (upper & lower 95% CI) values of 145,657 (110,073 & 192,744) and 29,551 (6,597 & 132,372). Interestingly, the SLA/LA+OVA formulation induced the second highest measured titers of 93,882 ± 10,845. In contrast, alum or CpG-adjuvanted vaccines induced the lowest levels of anti-OVA IgG with GMT (upper & lower 95% CI) values of 4489 (2,351 & 8,571) and 2266 (1,323 & 3,880), respectively. The anti-OVA IgG titers obtained with both archaeosome formulations were significantly greater than those observed with any of the other tested adjuvants (p<0.05). The only exception was SLA/LA vs. Montanide 720 where the differences between these two adjuvants did not reach a level of statistical significance.

When IgG isotypes (IgG1 and IgG2c), were measured, OVA adjuvanted with the Th2-biased adjuvants alum and Addavax induced the highest IgG1/IgG2c ratios of 415.9 and 132.4, respectively, while the Th1-biased adjuvants CpG and Poly(I:C) induced the most IgG2c-biased response, with measured IgG1/IgG2c ratios < 1 (Table 2). A more mixed IgG1/IgG2c response was induced by Montanide 720, alum/CpG, MPLA adjuvanted vaccines with IgG1/IgG2c ratios of 1.4, 4.5, 5.2, respectively whereas all three archaeosome formulations gave slightly IgG1-biased responses with IgG1/IgG2c ratios of 10.9 and 13.9 for SLA and SLA/LA archaeosomes, respectively. It was not possible to determine the IgG1/IgG2c ratio for OVA in the absence of any vaccine adjuvants since IgG titers were too low.

**Table 2:** OVA-specific IgG1 and IgG2c Ab titers.

Adjuvant	IgG1 (GMT)	IgG2c (GMT)	IgG1/IgG2c	
			GM	95% CI
**None**	100	100	1.0	1–1
**SLA**	360,435	53,882	10.9	4.1–10.8
**SLA/LA**	278,233	16,525	13.9	6.1–27.1
**Alum**	42,448	102	415.9	256.7–673.9
**CpG**	288	2,548	0.1	0.0–0.5
**Alum/CpG**	29,001	6,429	4.5	1.1–18.4
**Poly(I:C)**	24,708	36,264	0.6	0.2–1.7
**MPLA**	46,522	6,449	5.2	1.7–16.2
**Montanide 720**	42,057	21,319	1.4	1.0–4.1
**Addavax**	46,172	349	132.4	22.0–797.3

C57BL/6 mice (n = 10/group) were immunized i.m. with ovalbumin (20 μg) with or without adjuvant on days 0 and 21. Animals were bled on Day 28 and serum analyzed for anti-OVA IgG1 and IgG2c Ab titers. The titers were then used to calculate the IgG1 to IgG2c ratio per animal. The geometric mean titers (GMT) per group are presented, along with the geometric mean (GM) and lower to upper limits of the 95% confidence interval (CI) for the IgG1/IgG2c ratios.

### Cellular response to OVA vaccine formulations in mice

**Fig 2:**
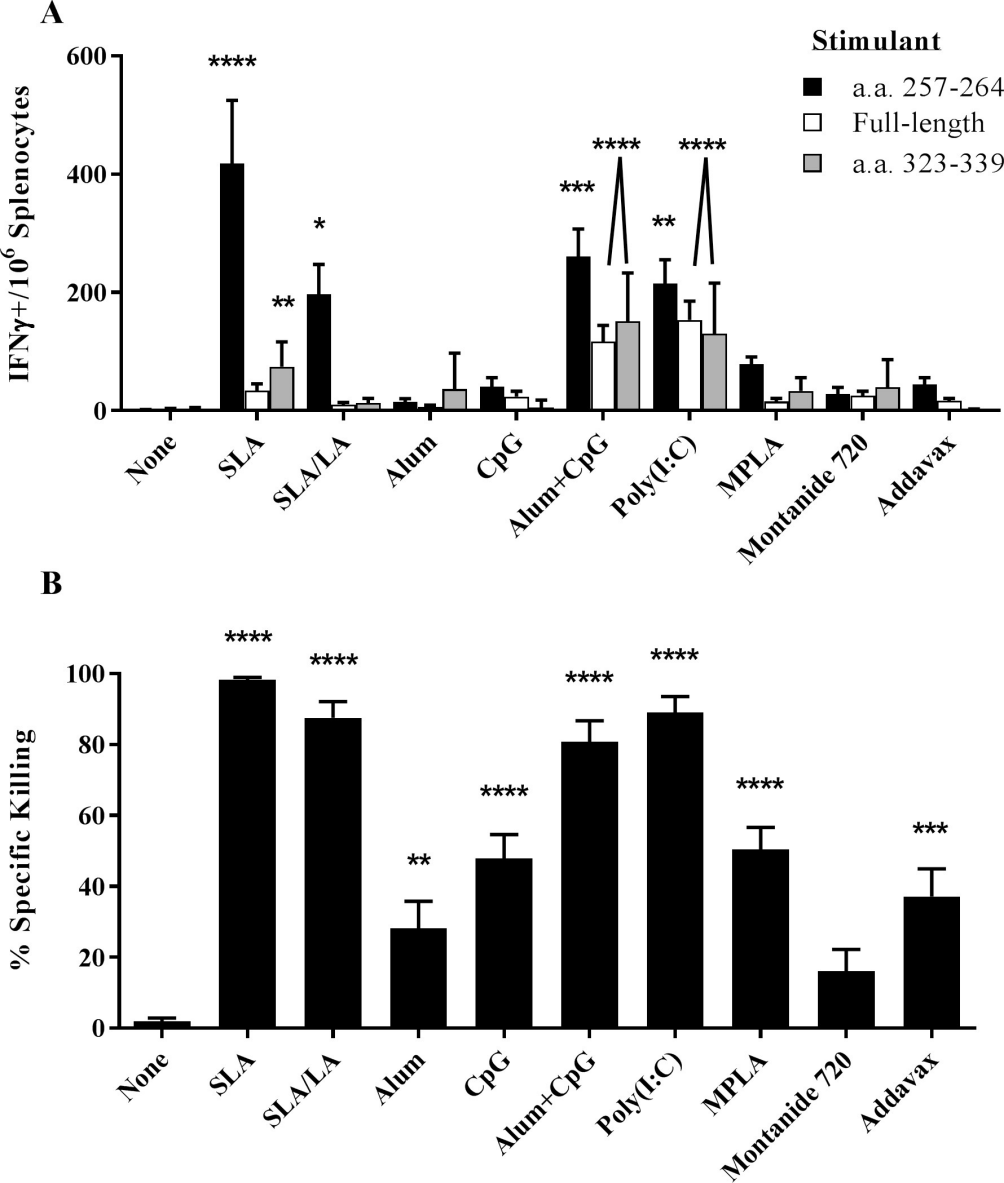
OVA-specific cellular immune responses in immunized mice. C57BL/6 mice (n = 10/group) were injected i.m. on Days 0 and 21 with ovalbumin (20 μg) with or without adjuvant. Splenocytes were harvested on Day 28 and analyzed by IFN-γ ELISpot when stimulated by OVA-specific peptides or protein (Panel A). In addition, the *in vivo* cytolytic activity of OVA-specific CD8^+^ cells was assessed by the measurement of CFSE-labeled target cells in the splenocytes collected on Day 28 (Panel B). Grouped data is presented as mean + standard error of mean (SEM). *: p<0.05, **: p<0.01, ***: p<0.001 & ****: p<0.0001 for various groups receiving adjuvanted formulations when compared to unadjuvanted control group by one-way ANOVA followed by Dunnett's multiple comparisons test.

OVA-specific T cell responses were assessed through IFNγ ELISpot and an *in vivo* CTL assay. Seven days following the second vaccination, splenocytes from mice immunized with OVA encapsulated in the two archaeosome formulations had significantly higher levels of IFN-γ+ T cells induced by a CD8 T cell specific peptide OVA_257-264_ than those obtained in the unadjuvanted group (p<0.0001 and p<0.05, with SLA and SLA/LA, respectively; Fig 2A). OVA adjuvanted with alum/CpG or Poly(I:C) were also strong inducers of OVA_257-264_ responsive T cells with a mean of 261 and 215 IFN-γ positive cells/10^6^ splenocytes, respectively (p<0.01 vs. unadjuvanted group). The SLA formulation induced the greatest amount of IFN-γ positive OVA-specific CD8 T cells, with 418 IFN-γ positive cells/10^6^ splenocytes which was ~2-fold greater than obtained with formulations adjuvanted with SLA/LA or Poly(I:C) (p<0.05). However, alum/CpG and Poly(I:C) adjuvanted vaccines induced more IFN-γ+ T cells specific to the CD4 epitope OVA_323-339_ than SLA. With this stimulant, alum/CpG, Poly(I:C) and SLA-adjuvanted vaccines induced significantly higher numbers of spots than mice immunized with OVA alone (p<0.0001 for alum/CpG and Poly(I:C), p<0.01 for SLA; average of 153, 116 and 75 IFN-γ positive cells/10^6^ splenocytes, respectively. Similar results were obtained when cells were stimulated with the full-length OVA protein (r^2^ = 0.514; S1A Fig). A much weaker correlation was obtained when comparing OVA_257-264_ vs. whole protein or OVA_257-264_ vs. OVA_323-339_, (r^2^ = 0.191 and 0.259, respectively; S1B & S1C Fig). OVA-specific killing of target cells coated with OVA_257-264_ was also measured using an *in vivo* CTL assay of the same splenocyte samples. All adjuvanted vaccine formulations, with the exception of OVA/Montanide 720, induced significantly higher killing than in control animals immunized with OVA alone (Fig 2B). Highest specific killing (99%) was induced by the SLA-adjuvanted formulation, which was significantly higher than obtained with alum, CpG, MPLA, Montanide 720 or Addavax adjuvanted OVA vaccines (p<0.0001). SLA/LA, Poly(I:C) and alum/CpG adjuvanted vaccines were also strong inducers of CTL activity yielding 80–85% killing of target cells. There was a strong correlation with the measured killing in the CTL assay and the ELISpot results with the SIINFEKL epitope (r^2^ = 0.437; S1D Fig).

### Humoral response to HBsAg vaccine formulations in mice

**Fig 3:**
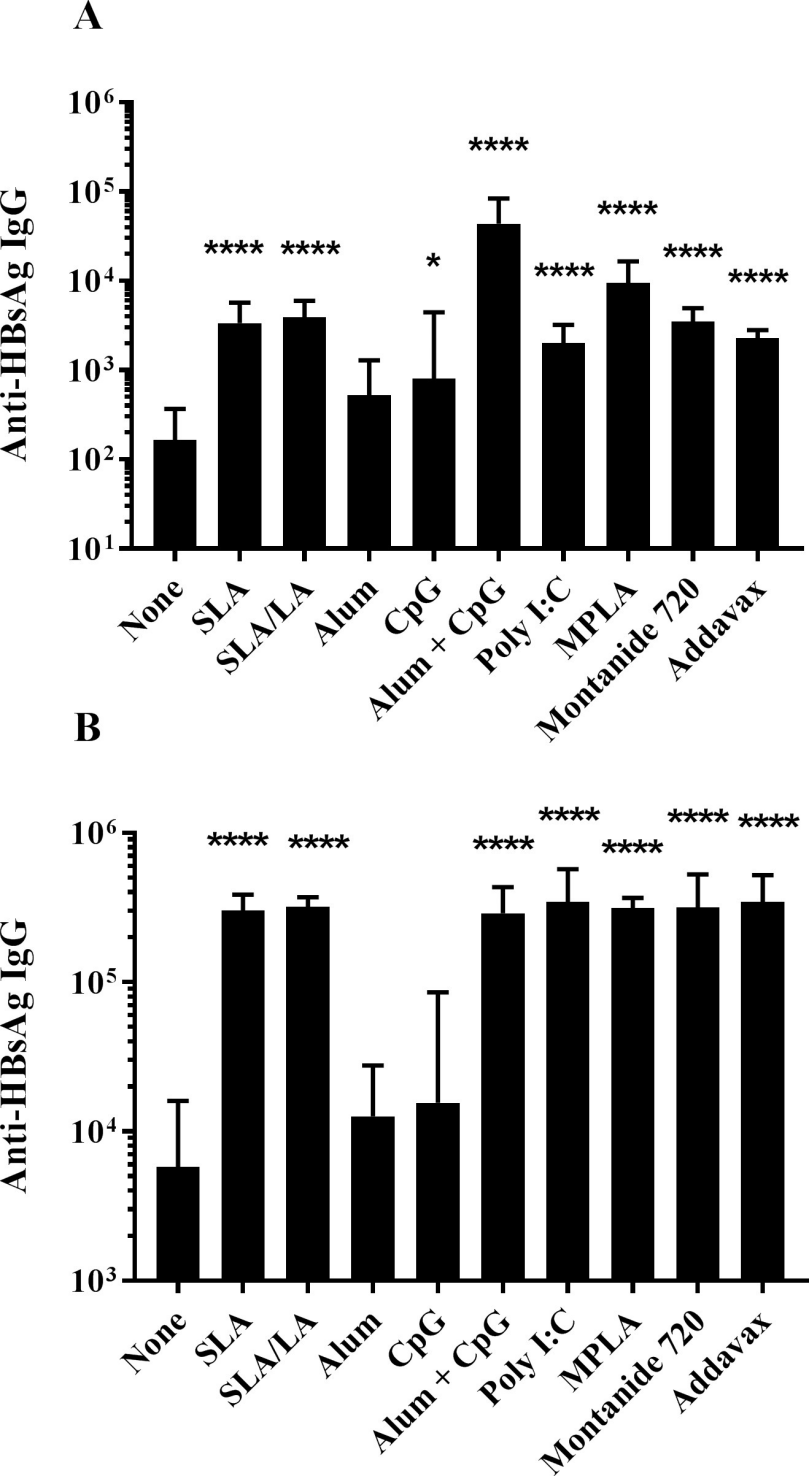
Anti-HBsAg IgG titers in immunized mice. BALB/c mice (n = 10/group) were immunized i.m. with HBsAg (2 μg) with or without adjuvant on days 0 and 21. Animals were bled on Day 20 (Panel A) and 28 (Panel B) with serum analyzed for anti-HBsAg IgG Abs by ELISA. Grouped data is presented as geometric mean titer (GMT) + 95% Confidence Interval (CI). *: p<0.05 & ****: p<0.0001 for various groups receiving adjuvanted formulations when compared to unadjuvanted control group by one-way ANOVA followed by Dunnett's multiple comparisons test.

BALB/c mice were immunized on days 0 and 21 with HBsAg alone or in combination with the same panel of adjuvants as tested with OVA. After a single vaccine dose, highest levels of anti-HBsAg IgG were induced using alum/CpG as adjuvant combination, with measured levels significantly higher than those obtained with all other tested formulations, except for MPLA + HBsAg (p<0.001; Fig 3A). All of the other adjuvanted vaccine formulations, except for alum alone, induced HBsAg-specific IgG titers greater than those induced with HBsAg alone (p<0.01). No significant differences were seen in the Ab titers induced by the archaeosome formulations compared to Poly(I:C), MPLA, Montanide 720 and Addavax. Post-second vaccine dose, all adjuvanted formulations, except for alum or CpG alone, induced significantly higher titers than antigen alone (p<0.0001; Fig 3B). No significant differences were observed between the titers obtained with SLA, SLA/LA, alum/CpG, MPLA, Poly(I:C), Addavax and Montanide 720, which were all significantly higher than those obtained with alum or CpG alone (p > 0.0001).

When IgG isotypes (IgG1 and IgG2a), were measured, HBsAg adjuvanted with alum and Montanide 720 induced the highest IgG1/IgG2a ratios of 79.8 and 92.0, respectively, whereas encapsulation of HBsAg in either of the archaeosome formulations or the addition of Addavax did not greatly alter the ratio of IgG1 to IgG2a compared to HBsAg alone (Table 3). In contrast, TLR agonists such as CpG, Poly(I:C) and MPLA pushed the response towards IgG2a, with average IgG1/IgG2a ratios of 0.4–0.8 observed.

**Table 3:** HBsAg-specific IgG1 and IgG2a Ab titers.

Adjuvant	IgG1 (GMT)	IgG2a (GMT)	IgG1/IgG2a	
			GM	95% CI
**None**	7,049	991	7.1	3.7–13.8
**SLA**	316,832	79,844	4.0	1.7–9.2
**SLA/LA**	314,803	102,519	3.1	1.4–6.6
**Alum**	26,410	331	79.8	30.9–206.3
**CpG**	8,904	11,685	0.8	0.4–1.4
**Alum/CpG**	190,606	133,205	1.4	0.8–2.5
**Poly(I:C)**	51,893	118,426	0.4	0.2–1.2
**MPLA**	62,075	129,727	0.5	0.1–1.5
**Montanide 720**	674,444	7,329	92.0	11.5–738.7
**Addavax**	607,476	62,299	9.8	4.8–19.9

BALB/c mice (n = 10/group) were immunized i.m. with HBsAg (2 μg) with or without adjuvant on days 0 and 21. Animals were bled on Day 28 and serum analyzed for anti-HBsAg IgG1 and IgG2a Ab titers. The titers were then used to calculate the IgG1 to IgG2a ratio per animal. The geometric mean titers (GMT) per group are presented, along with the geometric mean (GM) and lower to upper limits of the 95% confidence interval (CI) for the IgG1/IgG2c ratios.

### Cellular response to HBsAg vaccine formulations in mice

**Fig 4:**
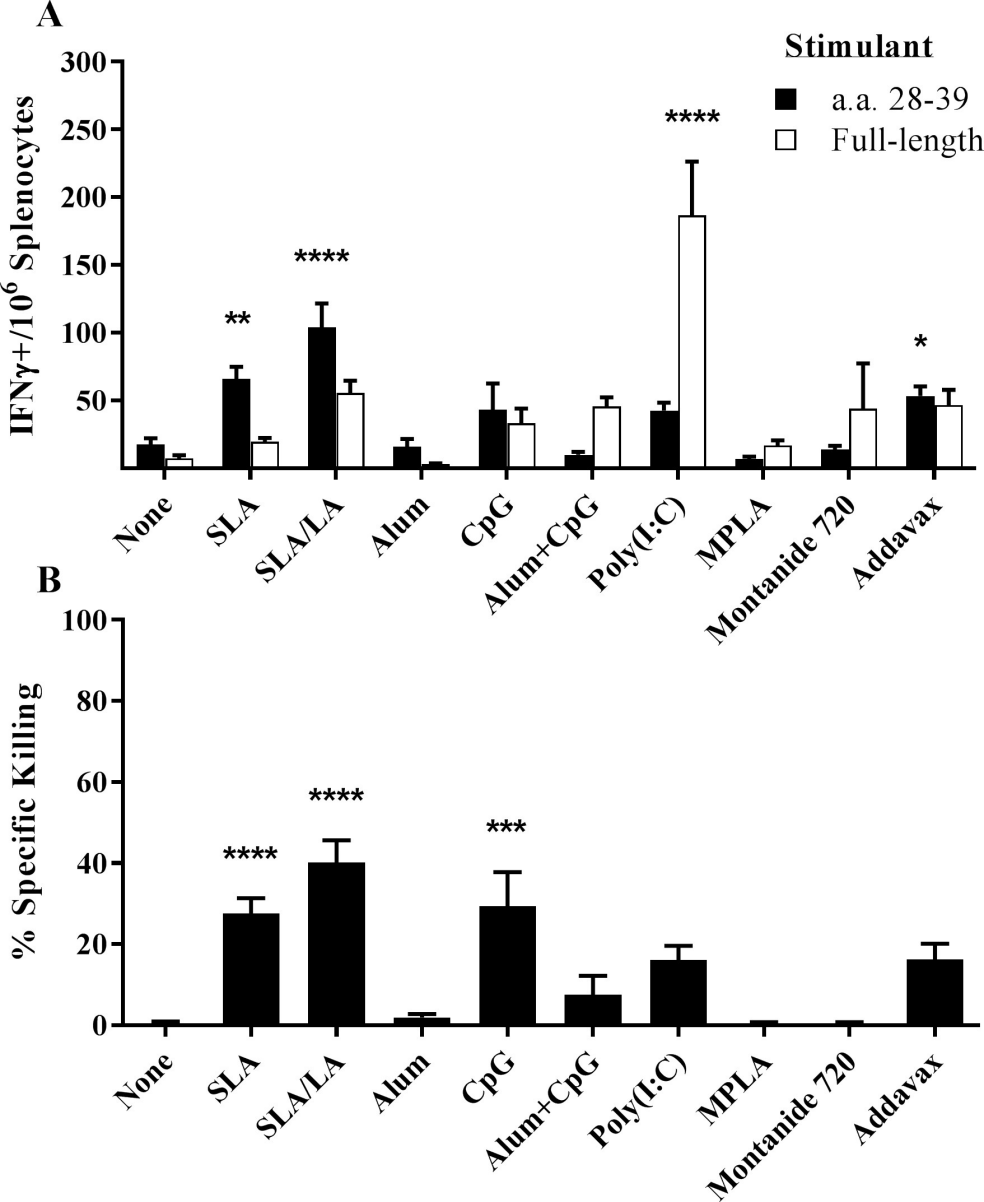
HBsAg-specific cellular immune responses in immunized mice. BALB/c mice (n = 10/group) were injected i.m. on Days 0 and 21 with HBsAg (2 μg) with or without adjuvant. Splenocytes were harvested on Day 28 and analyzed by IFN-γ ELISpot when stimulated by an HBsAg-specific peptide or full-length protein (Panel A). In addition, the *in vivo* cytolytic activity of HBsAg-specific CD8^+^ cells was assessed by the measurement of CFSE-labeled target cells in the splenocytes collected on Day 28 (Panel B). Grouped data is presented as mean + standard error of mean (SEM). *: p<0.05, **: p<0.01, ***: p<0.001 & ****: p<0.0001 for various groups receiving adjuvanted formulations when compared to unadjuvanted control group by one-way ANOVA followed by Dunnett's multiple comparisons test.

HBsAg-specific T cell responses were assessed using an IFNγ ELISpot and an *in vivo* CTL. For IFN-γ ELISpot analysis, a peptide corresponding to a previously identified CD8 T cell epitope (amino acid residues 28–39 of HBsAg) and the whole HBsAg protein were used. There was a significant increase (~4 to 5 -fold) in the number of IFN-γ+ splenocytes induced by HBsAg_28-39_ in mice immunized with HBsAg encapsulated in SLA (p<0.01) or SLA/LA (p<0.0001) archaeosomes compared to unadjuvanted HBsAg (Fig 4A). Of the other tested formulations, only Addavax-OVA induced a significant increase in HBsAg_28-39_-specific T cells over control mice injected with HBsAg alone (p<0.05). However, this was still significantly lower than that obtained with SLA/LA+HBsAg (p<0.01). Interestingly, only Poly(I:C)-adjuvanted HBsAg appeared to strongly induce T cells susceptible to stimulation with the whole HBsAg protein, with significantly higher numbers of IFN-γ+ cells seen as compared to all other groups (p<0.0001).

As with OVA, functionality of the HBsAg-specific CD8 T cells was evaluated by an *in vivo* CTL assay. Naïve splenocytes were pulsed with the HBsAg_28-39_ peptide to generate target cells prior to injection into immunized animals. Both archaeosome formulations induced killing of HBsAg-pulsed cells, with 28% and 40% killing seen in mice immunized with HBsAg encapsulated in SLA and SLA/LA archaeosomes (p<0.0001; Fig 4B), respectively. In addition, CpG alone induced a significant increase (29%) in killing (p<0.0001). There were no significant differences in CTL activity between these three formulations. As seen with OVA, there was a strong correlation obtained with the % killing and the number of IFN-γ+ splenocytes obtained when stimulated with the CD8 epitope (r^2^ = 0.615; S2A Fig), whereas correlation between % killing and IFN-γ+ splenocytes when stimulated with full-length HBsAg was quite weak (r^2^ = 0.018; S2B Fig).

### Induction of local cytokines/chemokines by vaccine formulations in mice

**Fig 5:**
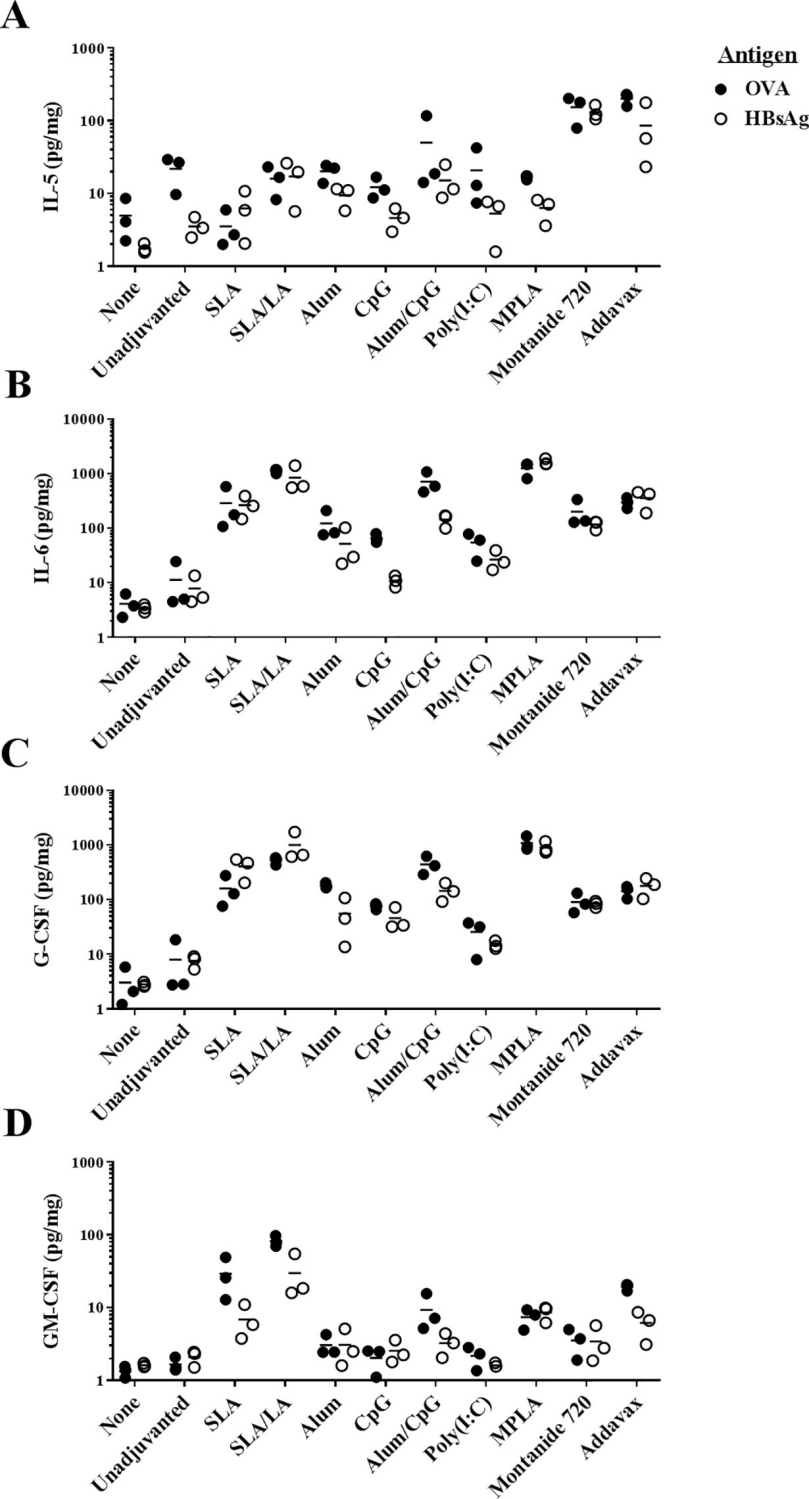
Cytokine levels at injection site after immunization in mice. C57BL/6 and BALB/c mice (n = 3/group) were injected i.m. with various OVA and HBsAg vaccine formulations, respectively. The levels of 17 different cytokines and chemokines were assessed in protein extracts derived from the left T.A. muscle collected 6 hours following injection. Levels of IL-5 (Panel A), IL-6 (Panel B), G-CSF (Panel C) or GM-CSF (Panel D) are shown.

The levels of 17 different cytokines/chemokines were measured in the vaccinated muscles 6 hours following immunization with the above mentioned vaccine formulations. Multiple adjuvants induced a strong inflammatory reaction when combined with either antigen (Tables 4 & 5). IL-6 levels were significantly higher in all adjuvanted groups with either antigen when compared to the unadjuvanted control (p<0.05), except for HBsAg + CpG. The strongest induction of IL-6 was seen with MPLA and SLA/LA with levels >99-fold higher when compared to antigen alone (Fig 5B). In the case of the Th2-biased cytokine IL-5, only the vaccine formulations incorporating the oil-in-water and water-in-oil emulsions, Addavax and Montanide 720, were shown to cause a significant increase in levels with both antigens (p<0.01; Fig 5A). The colony stimulating factor G-CSF was also strongly induced by multiple vaccine adjuvants, with significant increases measured following vaccination with all the adjuvanted vaccine formulations vs. the unadjuvanted control (p<0.05; Fig 5C), except for HBsAg+Poly(I:C). Again, the strongest induces of G-CSF were MPLA and SLA/LA, with a >65-fold increase in G-CSF levels found in the muscles as compared to antigen alone.

**Table 4:** Cytokine/Chemokine levels in mice after i.m. injection of OVA vaccine formulations.

Analyte	None	Unadju-vanted	SLA	SLA/LA	Alum	CpG	Alum/CpG	Poly(I:C)	MPLA	Montanide 720	Addavax
**IFN-γ**	3 (0)	4 (0)	4 (1)	3 (0)	4 (0)	4 (1)	5 (2)	4 (0)	3 (0)	3 (0)	3 (0)
**IL-1β**	6 (1)	7 (0)	11 (1)	**15 (1)**	9 (1)	10 (2)	**22 (6)**	9 (1)	**33 (6)**	8 (1)	13 (1)
**IL-2**	2 (0)	1 (0)	2 (0)	2 (0)	2 (0)	2 (0)	4 (1)	2 (0)	2 (0)	1 (0)	2 (0)
**IL-4**	1 (0)	1 (0)	2 (0)	**4 (1)**	1 (0)	1 (0)	**7 (3)**	1 (0)	1 (0)	2 (0)	**6 (1)**
**IL-5**	5 (2)	22 (6)	4 (1)	16 (4)	20 (3)	12 (2)	**50 (34)**	21 (11)	17 (1)	**154 (38)**	**202 (22)**
**IL-6**	4 (1)	11 (7)	289 (148)	**1130 (60)**	123 (44)	66 (7)	**714 (189)**	55 (16)	**1267 (227)**	201 (68)	299 (39)
**IL-10**	2 (0)	2 (0)	2 (0)	2 (0)	2 (0)	2 (0)	3 (1)	2 (0)	2 (0)	2 (0)	2 (0)
**IL-17**	1 (0)	2 (0)	2 (0)	1 (0)	2 (0)	1 (0)	2 (1)	1 (0)	1 (0)	1 (0)	1 (0)
**TNF-α**	2 (0)	2 (0)	3 (1)	3 (0)	2 (0)	**4 (1)**	3 (1)	2 (0)	**6 (1)**	1 (0)	2 (0)
**G-CSF**	3 (1)	8 (5)	160 (60)	**523 (47)**	181 (12)	76 (5)	**443 (97)**	26 (9)	**1086 (190)**	90 (22)	145 (21)
**GM-CSF**	1 (0)	2 (0)	**29 (11)**	**82 (8)**	3 (1)	2 (0)	9 (3)	2 (0)	7 (1)	4 (1)	**19 (1)**
**IP-10**	10 (1)	46 (10)	226 (27)	162 (23)	47 (2)	361 (52)	**567 (230)**	**1044 (154)**	**828 (220)**	24 (8)	46 (11)
**KC**	24 (11)	56 (23)	990 (304)	**1383 (68)**	299 (35)	133 (45)	**1282 (561)**	97 (28)	**1253 (85)**	231 (9)	527 (66)
**MCP-1**	40 (22)	186 (41)	**1621 (321)**	**1520 (114)**	342 (22)	410 (21)	1082 (498)	622 (140)	**1112 (48)**	487 (16)	981 (94)
**MIP-1α**	4 (1)	5 (1)	78 (18)	56 (8)	15 (1)	70 (6)	**147 (53)**	28 (5)	**219 (28)**	58 (1)	**162 (19)**
**MIP-1β**	0 (0)	4 (3)	147 (26)	126 (17)	23 (5)	146 (16)	**330 (115)**	89 (7)	**566 (85)**	121 (7)	**336 (44)**
**MIP-2**	11 (1)	22 (11)	491 (125)	660 (34)	154 (15)	105 (43)	**1333 (341)**	94 (58)	**1836 (334)**	438 (27)	**1111 (108)**

C57BL/6 mice (n = 3/group) were injected i.m. with PBS (no vaccine) or vaccine formulations containing OVA (20 μg) with or without adjuvant. The levels of 17 immune-related proteins were assessed in protein extracts derived from T.A. muscles 6 hours following injection. Average levels (pg/mg) per group are shown with standard error of mean (SEM) in brackets. Values underlined in bold indicate the measured levels ranked in the top three for a particular analyte amongst the various groups tested and were at least 2-fold higher than values obtained with unadjuvanted formulation.

**Table 5:** Cytokine/Chemokine levels in mice after i.m. injection of HBsAg vaccine formulations.

Analyte	None	Unadju-vanted	SLA	SLA/LA	Alum	CpG	Alum/CpG	Poly(I:C)	MPLA	Montanide 720	Addavax
**IFN-γ**	1 (0)	1 (0)	1 (0)	**3 (1)**	2 (0)	1 (0)	1 (0)	1 (0)	2 (1)	1 (0)	3 (1)
**IL-1β**	3 (0)	7 (1)	10 (2)	**18 (2)**	8 (2)	6 (1)	7 (1)	6 (0)	**53 (7)**	9 (2)	**17 (3)**
**IL-2**	2 (0)	3 (0)	3 (0)	4 (0)	3 (0)	3 (0)	3 (0)	3 (0)	3 (1)	3 (0)	3 (0)
**IL-4**	2 (0)	2 (0)	2 (0)	2 (0)	2 (0)	2 (0)	2 (0)	2 (0)	2 (0)	2 (0)	2 (0)
**IL-5**	2 (0)	4 (1)	6 (3)	**17 (6)**	9 (2)	5 (1)	15 (5)	5 (2)	6 (1)	**130 (18)**	**86 (47)**
**IL-6**	3 (0)	8 (3)	265 (70)	**857 (281)**	52 (26)	11 (1)	145 (23)	27 (7)	**1649 (122)**	118 (12)	**358 (84)**
**IL-10**	2 (0)	3 (0)	3 (1)	4 (0)	3 (1)	3 (0)	3 (0)	2 (0)	4 (0)	3 (1)	4 (0)
**IL-17**	1 (0)	1 (0)	1 (0)	1 (0)	1 (0)	1 (0)	1 (0)	1 (0)	1 (0)	1 (0)	1 (0)
**TNF-α**	2 (0)	2 (0)	**7 (0)**	**4 (1)**	2 (0)	2 (0)	2 (0)	2 (0)	**5 (1)**	2 (0)	**4 (1)**
**G-CSF**	3 (0)	8 (1)	**405 (103)**	**1003 (367)**	55 (28)	46 (13)	145 (32)	15 (1)	**903 (133)**	84 (6)	179 (41)
**GM-CSF**	2 (0)	2 (0)	7 (2)	**30 (13)**	3 (1)	3 (1)	3 (1)	2 (0)	**9 (1)**	3 (1)	**6 (2)**
**IP-10**	5 (2)	158 (47)	232 (71)	468 (146)	9 (1)	508 (101)	34 (9)	**983 (87)**	**887 (208)**	24 (4)	**734 (48)**
**KC**	8 (3)	21 (6)	690 (193)	**1139 (232)**	145 (59)	48 (5)	379 (74)	48 (13)	**1578 (293)**	140 (21)	**699 (98)**
**MCP-1**	18 (2)	192 (49)	**1887 (178)**	1167 (145)	258 (95)	436 (75)	480 (86)	705 (43)	**1208 (99)**	384 (38)	**1367 (100)**
**MIP-1α**	2 (0)	2 (0)	**70 (7)**	48 (4)	3 (1)	30 (8)	13 (5)	5 (0)	**199 (22)**	37 (3)	**250 (43)**
**MIP-1β**	1 (0)	10 (4)	**171 (14)**	136 (8)	6 (3)	113 (16)	58 (21)	44 (2)	**635 (65)**	109 (16)	**582 (92)**
**MIP-2**	2 (1)	11 (1)	393 (27)	**564 (114)**	40 (23)	15 (3)	246 (76)	13 (2)	**2467 (494)**	306 (58)	**1326 (372)**

BALB/c mice (n = 3/group) were injected i.m. with PBS (no vaccine) or vaccine formulations containing HBsAg (2 μg) with or without adjuvant. The levels of 17 immune-related proteins were assessed in protein extracts derived from T.A. muscles 6 hours following injection. Average levels (pg/mg) per group are shown with standard error of mean (SEM) in brackets. Values underlined in bold indicate the measured levels ranked in the top three for a particular analyte amongst the various groups and were at least 2-fold higher than values obtained with unadjuvanted formulation.

**Fig 6:**
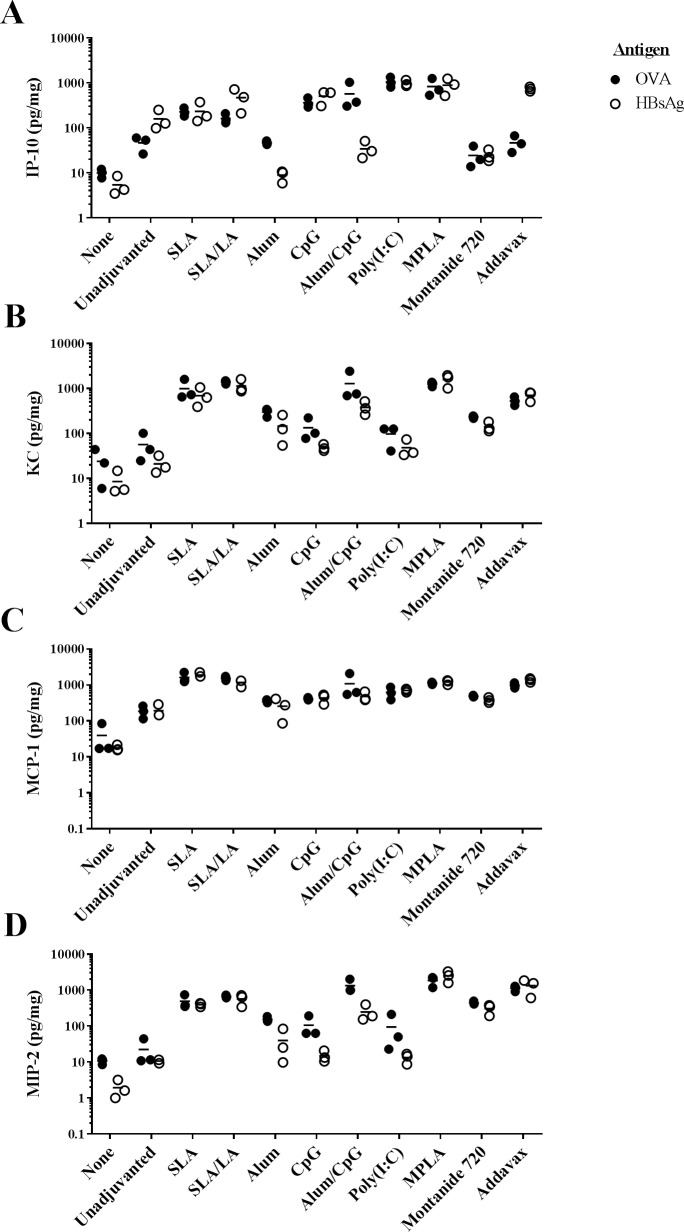
Chemokine levels at injection site after immunization in mice. C57BL/6 and BALB/c mice (n = 3/group) were injected i.m. with various OVA and HBsAg vaccine formulations, respectively. The levels of 17 different cytokines and chemokines were assessed in protein extracts derived from the left T.A. muscle collected 6 hours following injection. Levels of IP-10 (Panel A), KC (Panel B), MCP-1 (Panel C) or MIP-2 (Panel D) are shown.

Multiple chemokines were also strongly induced by the various vaccine formulations (Fig 6). Of note, levels of MIP-1α (CCL-3) and MIP-1β (CCL-4) at the injection site were significantly induced by all adjuvanted vaccine formulations with either antigen when compared to the unadjuvanted control, except for HBsAg+alum (p<0.05; Tables 4 & 5). MPLA and Addavax were the strongest inducers of these chemokines (30 to 139-fold increase over unadjuvanted vaccine formulation). MIP-2 (CXCL2) levels were also significantly increased (>20-fold) in formulations adjuvanted with SLA, SLA/LA, alum/CpG, MPLA, Addavax and Montanide 720 regardless of antigen when compared to unadjuvanted controls (p<0.0001; Fig 6D). Inclusion of alum, CpG or Poly(I:C) in the OVA vaccine formulations resulted in a relatively modest ~4-6-fold increase in MIP-2 levels (p<0.05), but no significant increase was seen when these adjuvants were combined with HBsAg. A similar trend was seen with KC (CXCL1), where all adjuvants, except for CpG and Poly(I:C), induced significant increases in the chemokine’s levels in the muscle when combined with either OVA or HBsAg (p<0.01; Fig 6B). SLA or SLA/LA archaeosomes were effective inducers of KC and MCP-1 (CCL2), with an average of 690–1383 and 1167–1887 pg/mg recorded in the muscles of mice immunized with the adjuvanted vaccine formulations vs. 21–56 and 186–192 pg/mg with antigen alone, respectively (Fig 6B & 6C; p<0.0001). In the case of IP-10 (CXCL10), certain adjuvants such as SLA/LA, CpG, Poly(I:C) and MPLA, induced an increase in chemokine levels when combined with either antigen (p<0.05), while other adjuvants appeared to have an antigen-specific inhibitory effect (Fig 6A). Significantly lower levels (~5-20-fold) of IP-10 were observed with HBsAg formulations adjuvanted with alum, alum/CpG or Montanide 720 vs. antigen alone (p<0.01). When combined with OVA, alum and Montanide 720-adjuvanted formulations induced similar levels of IP-10 as antigen alone, while alum/CpG induced a ~12-fold increase (p<0.0001).

## Discussion

Archaeosome-based adjuvants have been shown to induce robust and long-lasting humoral and cell-mediated immune responses to encapsulated antigen in multiple pre-clinical mice models, enhancing protection in murine models of infectious disease and cancer [23]. We have previously shown that SLA archaeosomes are well-tolerated in mice at doses 10-fold higher than generally used in a vaccine setting with no observed morbidity, altered body weights/temperatures or deviations in blood biochemistry/hematology parameters compared to control mice and that they induced cytokine production, immune cell trafficking and antigen uptake at the injection site [13]. However, only very limited studies have been conducted comparing archaeosomes with other adjuvants. For example, studies comparing TPL archaeosome formulations with alum or conventional liposomes showed that encapsulation of various antigens (bovine serum albumin, hen egg-white lysozyme and ovalbumin) within *M*. *smithii* TPL archaeosomes induced superior antigen-specific antibody responses and IFN-γ secretion than when antigen was combined with alum or conventional liposomes constructed with ester-based lipids [14]. To date, no comparative studies have been reported using the novel sulfated semi-synthetic SLA or SLA/LA archaeosomes. Therefore in this study we compared antigen-specific humoral and cellular immune responses induced to two different antigens (OVA and HBsAg) when adjuvanted with sulfated semi-synthetic archaeosomes, aluminum hydroxide, the squalene-based oil-in-water emulsion Addavax, the squalene-based water-in-oil emulsion Montanide 720, the TLR3 agonist Poly(I:C), the TLR4 agonist MPLA, and the TLR9 agonist CpG ODN. These adjuvants were selected as they are either approved for human use or in clinical testing, cover a range of different mechanisms of action and are readily available. Although not in clinical testing, Addavax was selected due its similarity to MF59, an approved squalene-based oil-in-water emulsion.

Alum based mineral salts have been used for many decades and are already in several licensed vaccines. Originally believed to augment immune responses by functioning as an antigen depot, the formation of NLRP3 inflammasomes is now thought to be a key mechanism associated with alum activity. Alum-containing vaccines lead to a release of many cytokines, such as IL-1β, IL-18 and IL-33, which are linked to the promotion of the Th2-type immune responses often associated with alum [1;24]. While immune responses with alum-adjuvanted vaccine formulations were higher than those obtained with unadjuvanted antigen in our study, they were relatively weak in comparison to those obtained with many of the other adjuvants, including the archaeosome-adjuvanted formulations, which generated significantly higher humoral and cell mediated immune responses to both OVA and HBsAg than obtained with alum as adjuvant.

We also compared the activity of our SLA and SLA/LA archaeosomes to Addavax, a squalene-based oil-in-water emulsion similar to MF59, and to Montanide 720, a squalene-based water-in-oil emulsion. MF59 has been licensed for use in Europe since 1997 and in the United States since 2015 for the influenza vaccine, Fluad. The safety of MF59 is now well-established and its mechanism of action has been extensively studies over the years [25–28]. MF59 leads to an increased expression of multiple factors linked to immune activation at the injection site, resulting in an enhanced production of chemoattractant cytokines, recruitment of immune cells to the injection site, enhanced antigen uptake and the transfer of small amounts of both antigen and adjuvant to draining lymph nodes. Herein, when Addavax was compared to SLA or SLA/LA archaeosomes, all three vaccine formulations induced strong antigen-specific IgG responses, which in the case of OVA was more Th2 biased based on a higher IgG1/IgG2a ratio when adjuvanted with Addavax than with the archaeosomes. Overall, stronger cell mediated immune responses, in particular with OVA, were induced by the archaeosome formulations than with Addavax. In contrast to MF59 and Addavax, Montanide 720 is a squalene-based water-in-oil emulsion [29], which when combined with antigen has been shown to induce high levels of antigen specific antibodies in many animal species [30]. In addition, it has been used in over 200 clinical trials targeting cancer, AIDS, malaria and autoimmune diseases and has a favorable safety profile [31]. In comparison to traditional soluble vaccines, water-in-oil emulsions allow for the slow release of antigen from the water droplets inside the oil, lengthening the exposure of immune cells to antigen. This drives sustained antibody production and continuous stimulation of cell-mediated responses. However, the continued interaction of antigen with activated T cells may also lead to T-cell tolerance and the proliferation of low-avidity CD8+ T cells [7;32–34]. In this study, both archaeosome formulations induced similarly strong antigen-specific antibody responses to the Montanide 720 adjuvanted vaccines. However, cell-mediated immune responses to OVA and HBsAg, as demonstrated by *in vivo* CTL and the levels of antigen-specific IFN-γ splenocytes, were markedly stronger following vaccination with the archaeosome formulations than with the Montanide 720-adjuvanted vaccines.

TLR agonists were developed as scientists gained a better understanding of how immune cells are activated through the interaction of pattern-recognition receptors (PRR) with pathogen-associated molecular patterns (PAMPs), “danger signals” found predominantly in bacteria and viruses [35]. PAMPs were integral components of the original live, attenuated or killed whole cell vaccines, but are largely absent in the currently developed highly purified subunit vaccines. Toll-like receptors (TLRs), a class of PRR found either on the cell surface or in endosomal compartments of multiple immune cells, bind ligands such as lipopolysaccharides (LPS), unmethylated CpG motifs or double-stranded RNA. Once activated, TLRs trigger cell signaling pathways and modulate gene expression through transcription factors such as NF-κB leading to increased levels of proinflammatory cytokines [36]. When mixed with antigen, synthetic TLR agonists can induce high levels of antigen-specific antibodies as well as strong cell-mediated immunity, which is traditionally absent or weak with alum-adjuvanted vaccines. For example, vaccines containing TLR/PRR agonists as adjuvants (i.e. CAF01, GLA-SE and IC31) promote high levels of IFN-γ+ antigen-specific cells *in vivo*, while alum and MF59 induce cytokines such as IL-5 [37]. Herein, we selected 3 different TLR agonists namely Poly(I:C) (TLR3 agonist, MPLA (TLR4 agonist) and CpG ODN (TLR9 agonist).

Administration of Poly(I:C), a synthetic double-stranded RNA (dsRNA) recognized by multiple PRRs, (e.g., TLR3, MDA-5 and RIG-I), leads to immune cell activation and the production of type I interferons *in vivo* [38;39]. It has been evaluated mainly as a cancer immunotherapy, either administered alone or as a vaccine adjuvant combined with tumor-associated antigens resulting in enhanced levels of antigen-specific CD8 T cells and reduced tumor growth [40]. While efficacious in multiple murine tumor models, it has not yet been approved for human use and, when evaluated as a monotherapy in patients with leukemia or solid tumors, has been associated with high toxicity, low activity and low stability at high doses [41]. In our study, Poly(I:C) did induce strong OVA- and HBsAg-specific antibody responses, however responses with SLA & SLA/LA encapsulated antigens were either superior (OVA) or equivalent (HBsAg). In the CD8 T cell associated readouts, the Poly(I:C) vaccine formulation induced similar or slightly lower levels of killing as SLA or SLA/LA encapsulated antigen in the *in vivo* CTL assay with OVA and HBsAg, respectively. Interestingly, Poly(I:C) appears to be an efficient inducer of antigen-specific CD4 T cell responses as measured by IFN-γ ELISpot. Poly(I:C) has previously been shown to enhance survival of CD4 T cells [42], and induce OVA_323-339_-specific T cells when administered with OVA antigen and anti-CD40 *in vivo* [43]. Longhi *et al*. have shown in head-to-head studies that Poly(I:C) was superior to other TLR agonists such as LPS and CpG in generating antigen-specific CD4 T cells when administered as an adjuvant with an experimental HIV vaccine [44]. This ability to generate antigen-specific CD4 T cells was linked to the adjuvant’s ability to induce high levels of type I interferons which in turn mediate dendritic cell maturation/activation and increased expression of CD4-interacting MHC class II. As type I interferons were not measured in our cytokine/chemokine panel (Tables 4 & 5), we are unable to confirm this link in our studies.

MPLA is an endotoxin-derived TLR4 agonist currently formulated in marketed vaccines such as Fendrix and Cervarix as part of the AS04 adjuvant system [7]. The use of MPLA is centered on its ability to enhance vaccine immune responses, while not exhibiting the high toxicity and low tolerability associated with LPS. While LPS activates expression of cytokines through MyD88 and TRIF signaling downstream from TLR4, MPLA appears to act more as a TRIF-biased agonist [45]. Despite this more restricted activation profile, MPLA-adjuvanted vaccines are capable of inducing antigen-specific CD4 and CD8 T cells. As expected, MPLA did induce strong antigen-specific antibody responses to both OVA and HBsAg. As with Poly(I:C), responses were either equivalent (HBsAg) or inferior (OVA) to those obtained with SLA or SLA/LA-encapsulated antigens. While MPLA did induce cytotoxic CD8 T cell activity when administered with OVA, it did not induce measurable levels of antigen-specific T cells when formulated with HBsAg. In kidney transplant recipients who did not respond to a 1^st^-generation alum-adjuvanted hepatitis B vaccine, immunization with the MPLA-adjuvanted Fendrix vaccine induced protective antibody titers in most patients, but antigen-specific cellular responses as assessed by IFN-γ ELISpot were not detected in 16/17 patients [46].

CpG oligonucleotides contain unmethylated cytosine-guanine tandem sequences characteristic of prokaryotic DNA that are recognized by TLR9 [47]. These synthetic oligonucleotides fall into 3 separate classes based on their activation profile and structural features: A-, B- and C- class. The recently approved 2^nd^ generation Hepatitis B vaccine, Heplisav-B, containing the 1018 ISS B-class oligonucleotide, was shown to induce strong antigen-specific antibody titers in patient populations that generally respond poorly to alum-adjuvanted vaccines [8]. A 3^rd^-generation anthrax vaccine, NuThrax, containing alum and the B-class oligonucleotide CPG 7909 is currently undergoing development with approval expected in the near future [48]. Our studies evaluating the murine B class CpG ODN 1826, showed that it could induce strong antigen-specific antibody responses to both OVA and HBsAg only when combined with alum. The inclusion of alum with CpG appears to enhance CD8 T cell responses with the OVA vaccine formulation, but not when administered with HBsAg. While generally CpG drives a Th1 biased immune response associated with CD8 T cell activity, alum is more geared towards to Th2 [1]. Also, as different mouse strains were used with each of the antigens, their bias towards Th1 (C57Bl/6) vs. Th2 (BALB/c) could contribute to the impact of alum on the Th1-driven immune response generated by CpG.

With the varying mechanisms of action mentioned above, it was interesting to directly compare the ability of the different adjuvants to generate an inflammatory milieu through the induction of cytokines/chemokines at the injection site shortly following vaccination and assess whether it correlated to their ability to augment antigen-specific humoral and cellular responses in the vaccine studies. Interestingly, vaccine formulations containing MPLA and Addavax induced large increases in the levels of multiple inflammatory factors at the time point with both antigens. Both these adjuvants effectively enhanced antibody responses in the above-described vaccine studies, and did generate moderate CTL activity when compared to SLA or SLA/LA. The semi-synthetic archaeosome formulations consistently enhanced the levels of IL-6, G-CSF, GM-CSF, KC, MCP-1, MIP-1α, MIP-1β and MIP-2 when combined with either OVA or HBsAg. This supports our previous report showing increased levels of these same proteins at the injection site following administration of empty SLA/LA archaeosomes without antigen [13]. The oil-in-water and water-in-oil emulsions, Addavax and Montanide 720, stood out in their ability to induce IL-5 when co-formulated with OVA or HBsAg. Previous reports with the oil-in-water emulsion MF59 have shown it to increase levels of IL-5 in the immunized muscle [25]. IP-10 secretion is a known marker of CpG activity [49] and was shown to be induced by our OVA and HBsAg vaccine formulations containing CpG. Interestingly, the inclusion of alum with CpG inhibited the induction of IP-10 with HBsAg, but not with OVA. This correlates with the observations in our vaccine studies, where inclusion of alum with CpG enhanced and diminished cellular responses to OVA and HBsAg, respectively. In this study, different preparations of the archaeosome formulations were used than those administered in the vaccine study. Differences in size, zeta potential and antigen:lipid ratio, especially for the OVA formulations, were observed (Table 1). While the different formulation characteristics fell within acceptable parameters previously established as capable of enhancing antigen immunogenicity, it is possible that the different preparations would stimulate the immune system differently. In addition, cytokines/chemokines were only evaluated at a single time-point (6 hrs) post administration which may not have been optimal for all cytokines. It is also possible that the kinetics of cytokine/chemokine production varies with different adjuvants. It would be of interest in future studies to evaluate the kinetics of cytokine/chemokine production induced by SLA relative to other adjuvants and correlate this with their different mechanisms of actions. Also, it would be important to look at the impact of various archaeosome characteristics on its immunostimulatory potential.

Overall, we found that SLA and SLA/LA were effective adjuvants generating strong cellular and humoral antigen-specific responses to both antigens. As we have previously found, archaeosomes composed of either SLA individually or mixed with uncharged glycolipid (lactosylarchaeol) were effective carriers for encapsulated antigen and gave largely equivalent responses [12]. SLA and SLA/LA archaeosomes gave strong humoral and cell mediated responses with both antigens tested highlighting their strong immunostimulatory potential. In contrast, Poly(I:C) and alum/CpG generated strong CD8 T cell responses to OVA but they did not generate strong CTL activity or IFN-γ+ splenocytes with HBsAg, whereas Montanide 720-adjuvanted formulations induced strong antigen-specific antibody responses, but only weak CTL activity to either antigen. In addition, differences were observed in the ability of the adjuvants to induce CD4 T cell responses specific to OVA or HBsAg. Alum/CpG and Poly(I:C) induced significantly higher numbers of IFN-γ+ splenocytes after stimulation by the OVA CD4 epitope (a.a. 323–339) or OVA whole protein. Meanwhile, only Poly(I:C) was able to induce a significant increase in the levels of IFN-γ+ splenocytes reactive to HBsAg whole-protein. Antigen-specific CD4 T cells can be important mediators of vaccine function as they have been shown to contribute to the generation and survival of cytolytic CD8 T cells, and directly contribute to the efficacy of experimental vaccines in certain disease models such as *Leishmania* [50]. At the molecular level, we have shown that vaccine formulations containing SLA or SLA/LA consistently induce high levels of multiple cytokines and chemokines at the injection site. We have previously shown that SLA/LA archaeosomes impact the local immune micro-environment when administered intramuscularly *in vivo* to mice, stimulating immune cell recruitment (e.g. neutrophils, macrophages), enhanced antigen uptake/retention at the vaccination site and trafficking to local draining lymph nodes [13]. TPL-based archaeosomes were shown to enable antigen cross-presentation to MHC class I pathway [51]. While the exact mechanism of action for SLA archaeosome-based adjuvants remains to be elucidated, they clearly generate strong localized pro-inflammatory activity at the vaccination site which can contribute to their strong adjuvant activity. Further studies are ongoing to clarify the pathways and/or cell types through which SLA-based archaeosomes are acting.

In summary, we have demonstrated the ability of sulfated semi-synthetic archaeosome formulations to induce strong humoral and cellular immune responses to different encapsulated antigens. Their ability to induce antigen-specific responses was superior to many of the tested adjuvants, warranting further development and characterization of this unique adjuvant system.

## Supporting information

S1 Fig Correlation of ELISpot and *in vivo* CTL responses in OVA-immunized mice. (TIF)

S2 Fig Correlation of ELISpot and *in vivo* CTL responses in HBsAg-immunized mice. (TIF)

S1 Dataset Raw data for Figs 1–4 & Tables 2–5. (XLSX)
