# Supplementary figures and images for: Sulfated archaeol glycolipids: Comparison with other immunological adjuvants in mice
Source: PLoS One. 2018 Dec 4;13(12):e0208067. doi: 10.1371/journal.pone.0208067 (PMC6279041; doi:10.1371/journal.pone.0208067)

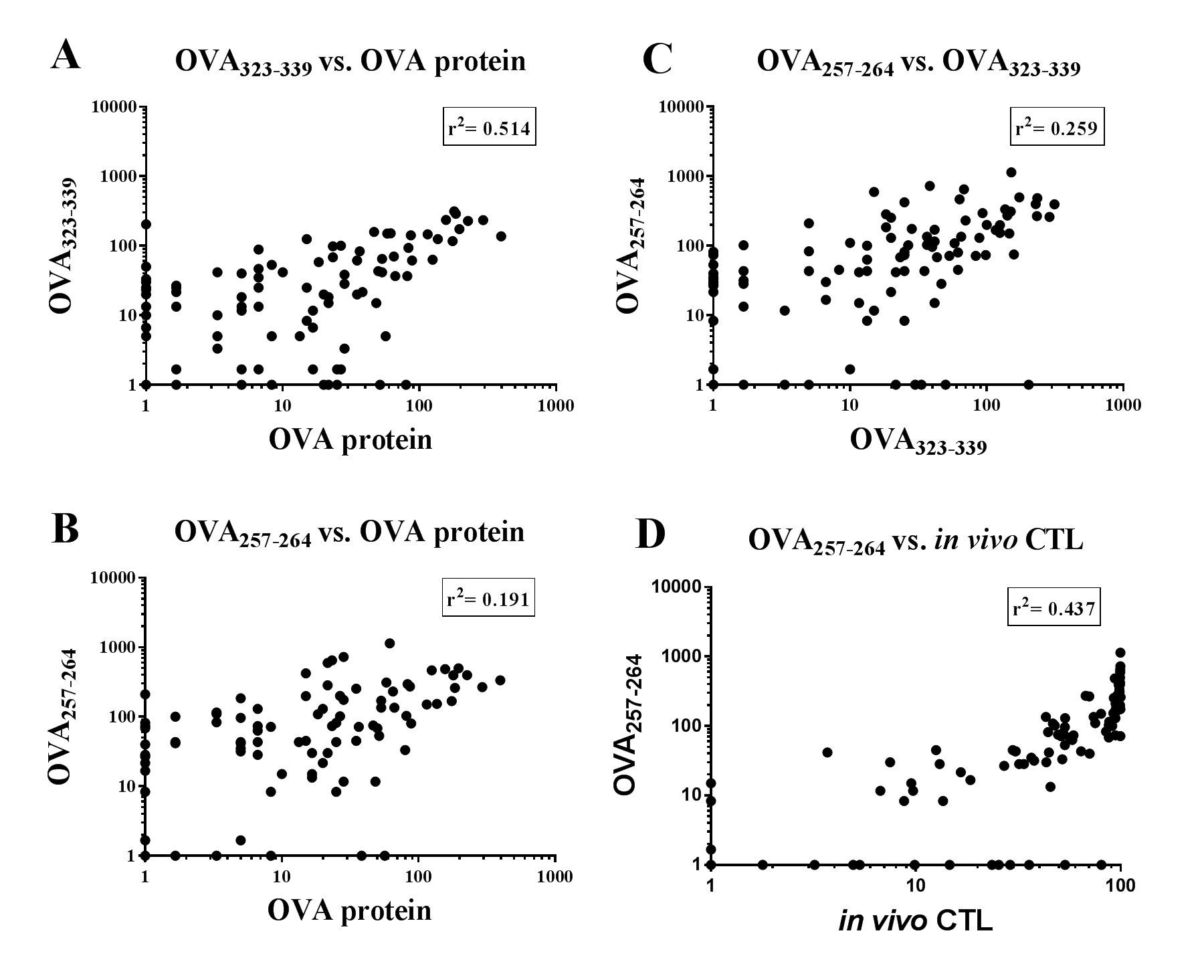

Supplement: S1 Fig — (TIF) [file pone.0208067.s001.tif]

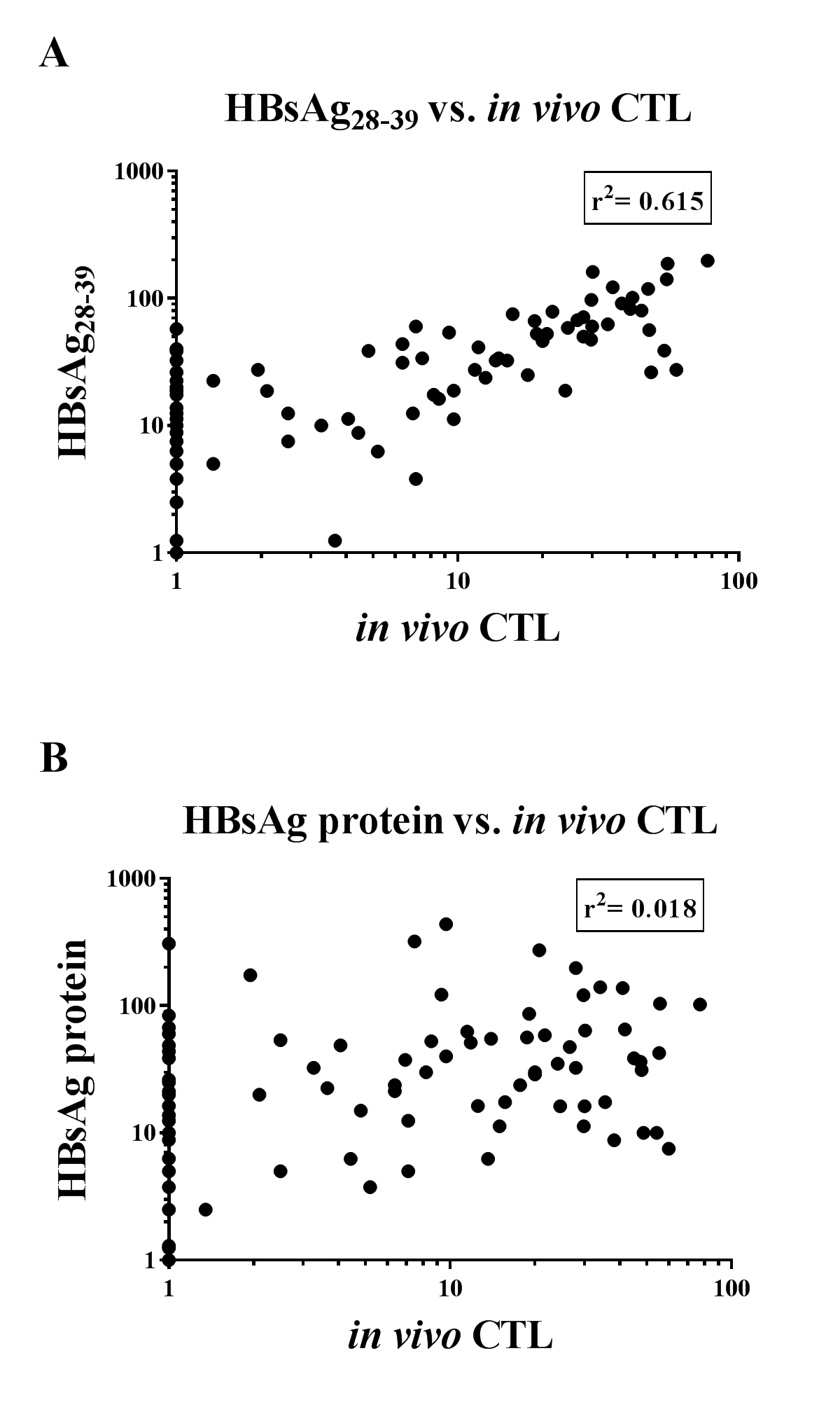

Supplement: S2 Fig — (TIF) [file pone.0208067.s002.tif]
